# Identification of Smith–Magenis syndrome cases through an experimental evaluation of machine learning methods

**DOI:** 10.3389/fncom.2024.1357607

**Published:** 2024-03-22

**Authors:** Raúl Fernández-Ruiz, Esther Núñez-Vidal, Irene Hidalgo-delaguía, Elena Garayzábal-Heinze, Agustín Álvarez-Marquina, Rafael Martínez-Olalla, Daniel Palacios-Alonso

**Affiliations:** ^1^Escuela Técnica Superior de Ingeniería Informática, Universidad Rey Juan Carlos, Madrid, Spain; ^2^Departament of Spanish Language and Theory of Literature, Universidad Complutense de Madrid, Madrid, Spain; ^3^Departament of Linguistics, Universidad Autónoma de Madrid, Madrid, Spain; ^4^Center for Biomedical Technology, Universidad Politécnica de Madrid, Madrid, Spain

**Keywords:** Smith–Magenis syndrome, machine learning, cepstral peak prominence, acoustics, children

## Abstract

This research work introduces a novel, nonintrusive method for the automatic identification of Smith–Magenis syndrome, traditionally studied through genetic markers. The method utilizes cepstral peak prominence and various machine learning techniques, relying on a single metric computed by the research group. The performance of these techniques is evaluated across two case studies, each employing a unique data preprocessing approach. A proprietary data “windowing” technique is also developed to derive a more representative dataset. To address class imbalance in the dataset, the synthetic minority oversampling technique (SMOTE) is applied for data augmentation. The application of these preprocessing techniques has yielded promising results from a limited initial dataset. The study concludes that the k-nearest neighbors and linear discriminant analysis perform best, and that cepstral peak prominence is a promising measure for identifying Smith–Magenis syndrome.

## 1 Introduction

Over time, artificial intelligence (AI) has experienced substantial growth in a variety of scientific areas and disciplines (Górriz et al., 2020, 2023). In the medical field, AI has been used for disease diagnosis and treatment (Rother et al., 2015; Shen et al., 2017; Jia et al., 2018; Li et al., 2019; Zhang et al., 2019; Spiga et al., 2020), as well as for new drug research, since, in scientific research, AI accelerates data analysis and complex phenomena monitoring (Cifci and Hussain, 2018; Firouzi et al., 2018). The versatility and transformative potential of AI offers new possibilities in disease diagnosis. The origins of AI date back to the 1950s, with the development of the first neural network (machine learning), although its roots can be traced even further back in time, considering previous approaches such as Bayesian statistics or Markov chains, which share similar concepts. In the case of Parkinson’s disease, the authors of Ali et al. (2019) worked on phonation in combination with ML. The results were applicable to other diseases that, due to their low incidence in the population, are understudied and, consequently, underdiagnosed.

Patients face considerable challenges with dealing with underdiagnosed pathologies. The lack of early detection and limited information deprives them of timely, pathology-specific care, which is especially important for young patients. The use of AI techniques for early disease detection is an ongoing challenge. In this study, the focus is on determining the discriminatory as well as pathological characteristics of young patients’ voices. Acoustic phonation studies provide relevant speaker information that can be used to detect diseases such as Alzheimer’s dementia, Parkinson’s, and amyotrophic lateral sclerosis, among others, based on the biomechanical uniqueness of each individual. Such uniqueness is evident in the EWA-DB dataset, which focuses on Slovak speakers with Alzheimer’s and Parkinson’s diseases (Rusko et al., 2023), and a dataset that focuses on Spanish native speakers with Parkinson’s disease (Orozco-Arroyave et al., 2014), as well as recent acoustic studies on Alzheimer’s (Cai et al., 2023; Zolnoori et al., 2023) and Parkinson’s (Warule et al., 2023) diseases. In the 2021 study by Lee (2021), two types of neural network models were developed for dysphonia detection: a Feedforward Neural Network (FNN) and a Convolutional Neural Network (CNN). These models were designed to utilize Mel Frequency Cepstral Coefficients (MFCCs) for the detection process.

The determined laryngeal biomechanics, elastin deficiency in Williams syndrome (WS) or excess laryngeal tension in the case of Smith–Magenis syndrome (SMS) (Watts et al., 2008; Moore and Thibeault, 2012; Hidalgo-De la Guía et al., 2021b) discriminate these syndromes from others caused by neurological pathologies based on genetics (Antonell et al., 2006; Albertini et al., 2010; Hidalgo et al., 2018; Jeffery et al., 2018; Hidalgo-De la Guía et al., 2021a). Specifically, the voice profile of an SMS patient is determined by excess laryngeal and acute tension f0. These patients may also have a certain degree of dysphonia, which is observed in both children and adults. Likewise, there are studies that suggest that certain syndromes present characteristic alterations in the voice that give rise to specific vocal phenotypes (Edelman et al., 2007; Brendal et al., 2017; Linders et al., 2023).

SMS is a genetic disease that affects neurological development from the embryonic stage, specifically due to the alteration of the RAI1 gene, which is considered responsible for most of the clinical abnormalities observed in SMS individuals (Slager et al., 2003; Vlangos et al., 2003). Given its prevalence, i.e., 1:15,000–25,000 births (Greenberg et al., 1996; Elsea and Girirajan, 2008; Girirajan et al., 2009), SMS is considered a rare disease and, therefore, is underdetected.

It is more common to approach the problem of rare disease detection from areas other than genetics, where the fundamental focus has been on characterization. ML techniques have recently been implemented in rare disease research, including SMS. Bozhilova et al. (2023) identified different profiles of autism characteristics in genetic syndromes associated with some intellectual disability. SMS was among the 13 syndromes studied. The *Social Communication Questionnaire* was used to train a support vector machine (SVM) that achieved an overall precision of 55%. The main limitations of this work were that only social communication skill metrics were used and imbalanced sample sizes across groups. One of the main results seems to indicate that autistic individuals with genetic syndromes have different characteristics than those without any genetic syndrome. In Frassineti et al. (2021) different ML models were proposed to allow the automatic identification of four different diseases, including SMS. They made recordings of subjects and extracted 34 acoustic characteristics with Praat and 24 with BioVoice. The *cepstral peak prominence* (CPP) was not among the extracted characteristics. After the results achieved by BioVoice for SMS (true positive rate of 55.6% and false-negative rate of 44.4%), the authors suggested that the vowel /a/ is not sufficient for the definition of phenotypes. In an extension of their previous work, the same authors (Calà et al., 2023) incorporated the vowels /a/, /I/, and /u/, and introduced a new control group of normative individuals. Utilizing BioVoice, they extracted 77 acoustic features, excluding CPP, and organized the subjects into three distinct groups: pediatric subjects (age < 12), adult females, and adult males. Each group was treated independently, with a unique Machine Learning model generated for each. The results, obtained through a 10-fold cross validation, are presented as mean accuracy along with the standard deviation. The pediatric group achieved an accuracy of 87 ± 9%, adult women achieved 77 ± 19%, and men achieved 84 ± 17%. However, the outcomes appear inconclusive due to the high variability in measures such as precision, recall, and f-score.

This work compares different Machine Learning techniques for the detection of SMS in young people using audio samples, from which only the CPP is computed and extracted. In addition, a novel windowing method is proposed to improve the performance of the models. In addition, the SMOTE technique is used, aiming outcomes in precision rates above 85%. This approach proposes a non-invasive, low-cost, and rapid detection method with only one acoustic parameter, which contrasts with methods based on genetic techniques.

Unfortunately, it is difficult to compare medical research works, which used genetic techniques, with non-invasive SMS detection. Likewise, mathematical and computational approaches to this syndrome use acoustical features such as formants, shimmer, and jitter, among others. However, this study case aims to open the exploration of new ways to identify SMS individuals. The fact to use only one feature (CPP) allows faster models with lower computational performance. Therefore, the ultimate goal is to detect the syndrome early using this single feature.

This article is organized as follows. In the following section, the methods and materials are explained, the dataset structure and the “window” method are highlighted, and the ML methods used are briefly explained from a theoretical perspective. In Section 3, the results are included, and the model training and validation, as well as the approach and results of the case studies, are detailed. Next, in Section 4, the obtained results are discussed, and finally, the conclusions and future lines of work are proposed.

## 2 Materials and methods

### 2.1 Cepstral peak prominence

This research work is based on the use of the CPP as a discriminant measure for the identification of SMS (nonnormotypic) individuals compared to a control group of normotypic individuals. The CPP is an acoustic parameter that allows determining the degree of periodicity of a voice, showing the prominence of a cepstral peak that varies according to the periodicity of phonation. The more pronounced the peak is, the more harmonic a voice (Hidalgo-De la Guía et al., 2021b).

In the past decade, it has been found that the CPP presents a strong correlation with the degree of voice dysphonia (Peterson et al., 2013; Brinca et al., 2014). In fact, higher correlations were found between the CPP, and dysphonic voices compared to those of typical distortion parameters (Moers et al., 2012). Currently, the CPP is considered one of the best acoustic parameters for estimating the degree of vocal pathology. In addition, it has been found that the CPP in SMS individuals is low, which could be related to a possible relationship between the syndrome and laryngeal biomechanics (Hidalgo-De la Guía et al., 2021b).

In SMS, a dysphonic voice is one of the characteristics with the highest rate of appearance (Linders et al., 2023), and to achieve dysphonic voice detection in this study, the CPP is used. The CPP is calculated as follows.

1. The signal is segmented into overlapping fragments (1,024 samples 87.5% overlap). Each fragment is multiplied by a Hamming window function, and the fast Fourier transform (FFT) is calculated. Based on this calculated signal, the absolute value is found, and its logarithm is calculated. Finally, the inverse fast Fourier transform (IFFT) is performed on the previous result, and the real part is obtained. Thus, a set of frames is created in the cepstral domain.
  $$c=\mathrm{real}\left(\mathrm{IFFT}\left(\log\left(\mathrm{abs}\left(FFT\left(x.\times w\right)\right)\right)\right)\right)$$
  where c is the cepstrum vector, x the input signal vector, w is a vector with a Hamming window function and the operation × represents the sample-to-sample product of both vectors.
2. A smoothing filter (smoothing in the cepstral direction) is applied to each of the frames obtained in the cepstral domain. This filter is applied to eliminate spurious signal values while preserving the true cepstral peaks, thus avoiding cepstral peak detection errors.
  $$c_{f}\left[n\right]=\overset{l-1}{\underset{i=0}{\sum }}a_{i}c\left[n-i\right]$$
  where *cf.* is the value of the smoothed cepstrum, *a_i_* are the coefficients of the filter, and l = 7 is the length of the filter in samples.
3. The cepstrum is then limited between the quefrency values corresponding to the minimum (22 samples) and maximum (400 samples) fundamental periods expected for the range of vocal frequencies of the study population.
4. The maximum value of the previous signal (cepstral peak) is calculated, and the CPP is obtained as the difference between this maximum and the average of the rest of the signal.
  $$CPP\left[i\right]=\max_{T_{\text{min}}}^{T_{\text{max}}}\left(c_{fi}\right)-\frac{\sum_{j=T_{\text{min}}}^{T_{\text{max}}}c_{fi}\left[j\right]-\max_{T_{\text{min}}}^{T_{\text{max}}}\left(c_{fi}\right)}{T_{\text{max}}-T_{\text{min}}}$$
5. A vector is formed with the CPP values thus obtained (CPP[n]), which is smoothed by a filter with a 56 ms window (smoothing in the temporal direction). This smoothing operation reduces the noise of the signal obtained while preserving large variations in the CPP value, which can be present in dysphonic voices.
  $$CPP_{f}\left[n\right]=\overset{m-1}{\underset{i=0}{\sum }}a_{i}CPP\left[n-i\right]$$
  with a filter length *m* = 7 for a displacement of 128 samples and 16,000 Hz of sampling frequency, and where ai are the coefficients of the filter (following a hamming window function), and CPPf the smoothed CPP.

### 2.2 Dataset

Most rare disease databases, such as those for SMS, are private, and accessing these databases is difficult. In the specific case of databases in Spanish, the Orphanet website (Orphanet, 2023) offers genetic biobank searches. Such searches were carried out, and three results were obtained: Basque Biobank, CIBERER Biobank, and the National Biobank for Rare Diseases (BioNer). However, two of the three results do not have information about SMS, and the one that does contain genetic information.

The difficulty of obtaining this type of data is well known. Given that the number of subjects suffering from these syndromes is small and heterogeneous, the datasets are strongly unbalanced. Consequently, this situation requires synthetic data augmentation methods to be applied. These techniques have been widely used in the field of image processing since the appearance of convolutional neural networks (CNNs) in 2012 (Shorten and Khoshgoftaar, 2019). Likewise, to process data such as those mentioned above, oversampling techniques such as the *synthetic minority oversampling technique* (SMOTE) and its variants are used. As described by Alabi et al. (2020), these techniques can be used to increase of amount of data in early tongue cancer detection. In Joloudari et al. (2023), the effectiveness of different solutions to data imbalance in Deep Neural Networks and CNNs is verified. The best result is obtained by combining SMOTE with a CNN plus a normalization process between both stages, achieving an accuracy of 99.08% across 24 imbalanced datasets.

In this study, the dataset contains voice quality information from normotypic and nonnormotypic individuals for comparison. To create this dataset, we worked with a total of 22 individuals between the ages of 5 and 33 who belong to the Smith–Magenis Spain Association (ASME), comprising 20% of the Spanish population diagnosed with this syndrome. The diagnosis of all the individuals with SMS was obtained by means of the fluorescent *in situ* hybridization (FISH) technique. Samples were collected from subjects through recordings in which they had to hold the vowel /a/ for a few seconds (minimum 500 ms of phonation). The recording quality was guaranteed by ruling out comorbidity of associated vocal pathology, such as vocal fold nodules or any other additional vocal problem. Likewise, the recording context was addressed as follows: the rooms were completely silent (some soundproofed), only of the researcher and the diagnosed person were in the room, and a cardioid lapel microphone was used. From all the audio, the CPP information, an acoustic voice quality measure and one of the best dysphonia metrics (vocal timbre alteration), as described by Heman-Ackah et al. (2003), was extracted.

In this study, a subset of these data was used, consisting of 12 individuals SMS, all of whom were between the ages of 5 and 12 years. These individuals were used because we wanted to verify the possibility of developing a system that allows early disease identification, since a late diagnosis leads to a worse quality of life. The group of 12 individuals with SMS is made up of two subgroups: a group of young children aged 5 to 7 years and another group of older children aged 8 to 12 years. Both subgroups had 3 boys and 3 girls.

To complete the dataset, 12 recordings of participants with typical development were added. Sample collection from normotypic individuals was the same as that used for SMS individuals, and the same age distribution as that of the SMS individuals was followed.

The dataset in the study contains 2,685 CPP values extracted from audio from the 24 participants (12 normotypic and 12 nonnormotypic participants). The number of CPP values per participant varied in relation to the number of voice samples obtained and their duration. Each entry in the dataset has the following fields defined: subject identifier, sex, age, CPP value, as well as whether the participant suffers from SMS and whether they belong to the “younger” or “older” group.

**Figure 1:**
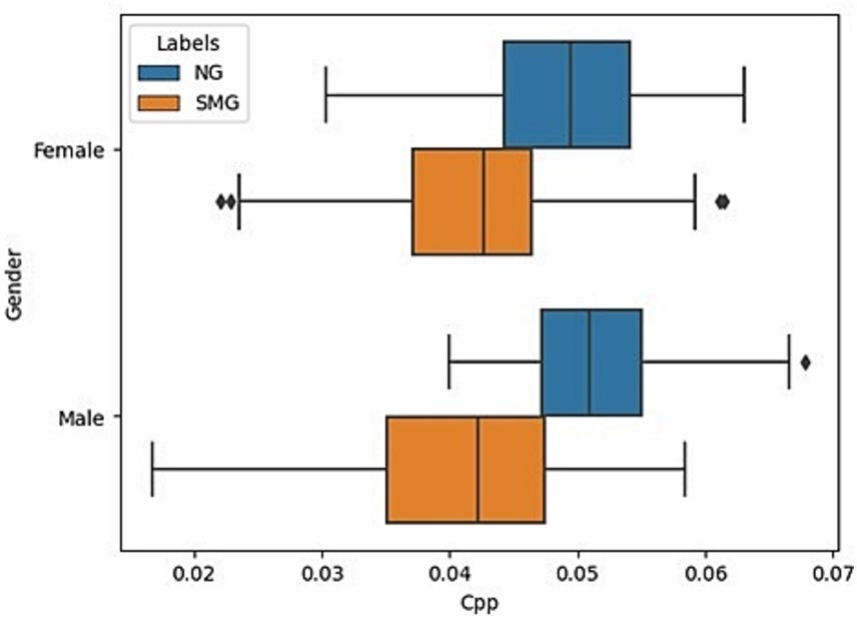
Representation of CPP values by sex, comparing normative vs. nonnormative groups.

A descriptive analysis of the CPP stored in the database was prepared as presented in Figure 1, where the *X*-axis represents the CPP values, and the *Y*-axis represents the data divided by sex. The orange boxplots represent the SMS group, and the blue boxplots represent the normative group. It is observed that the SMS group has much lower CPP values than those of the normative group. Likewise, it can be observed that the range of values for normative boys and girls is very similar. However, the range of values for SMS boys is slightly more dispersed than that of SMS girls. Finally, in Figure 1, it is observed that the *boxplot* of SMS girls is slightly larger, and the whiskers are somewhat longer than those of normotypic girls.

**Figure 2:**
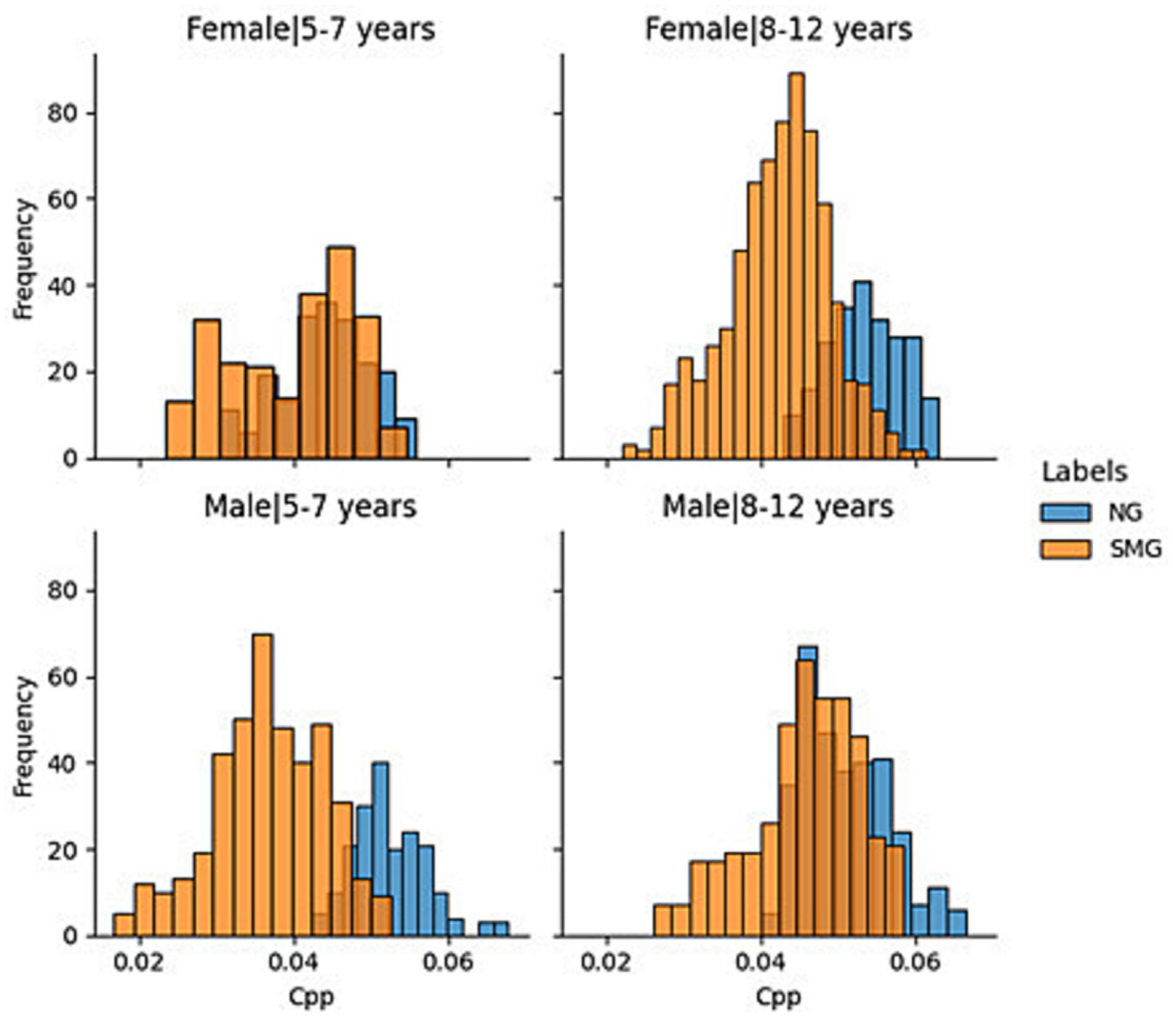
Normotypic vs. nonnormotypic CPP decomposition by sex and group.

Given the importance of age and sex and to improve the explainability of the results, the aforementioned information was segmented by “young children” (5–7 years) and “older children” (8–12 years). The results are reflected in Figure 2. From the generated histograms, it is observed that in the group of girls between 8 and 12 years old and that of boys between 5 and 7, there is a greater differentiation in the CPP values between normotypic and SMS individuals. However, in the other two groups (girls between 5 and 7 years old and boys between 8 and 12 years old), there is a greater overlap between the data of both groups. Specifically, the overlap is greater in girls between 5 and 7 years old than in the group of boys between 8 and 12 years old.

It is important to point out some of the potential research gaps in this research work. A larger number of individuals with SMS could be enriching and it could avoid lead to biases by gender, age, or other characteristics. The second issue is the lack of exploration of different alternatives to SMOTE. There are different variants of this technique and other oversampling methods that could be implemented and could lead to better solutions. Finally, other ML methods could also be searched. All four methods used in this research work have a multitude of variants that may improve the performance of the baseline method. Regarding the problem of the number of individuals, as previously mentioned, it has been decided to use a subset of the data as a first approach due to the number of patients who suffer from this syndrome.

### 2.3 Preprocessing and data augmentation

**Figure 3:**
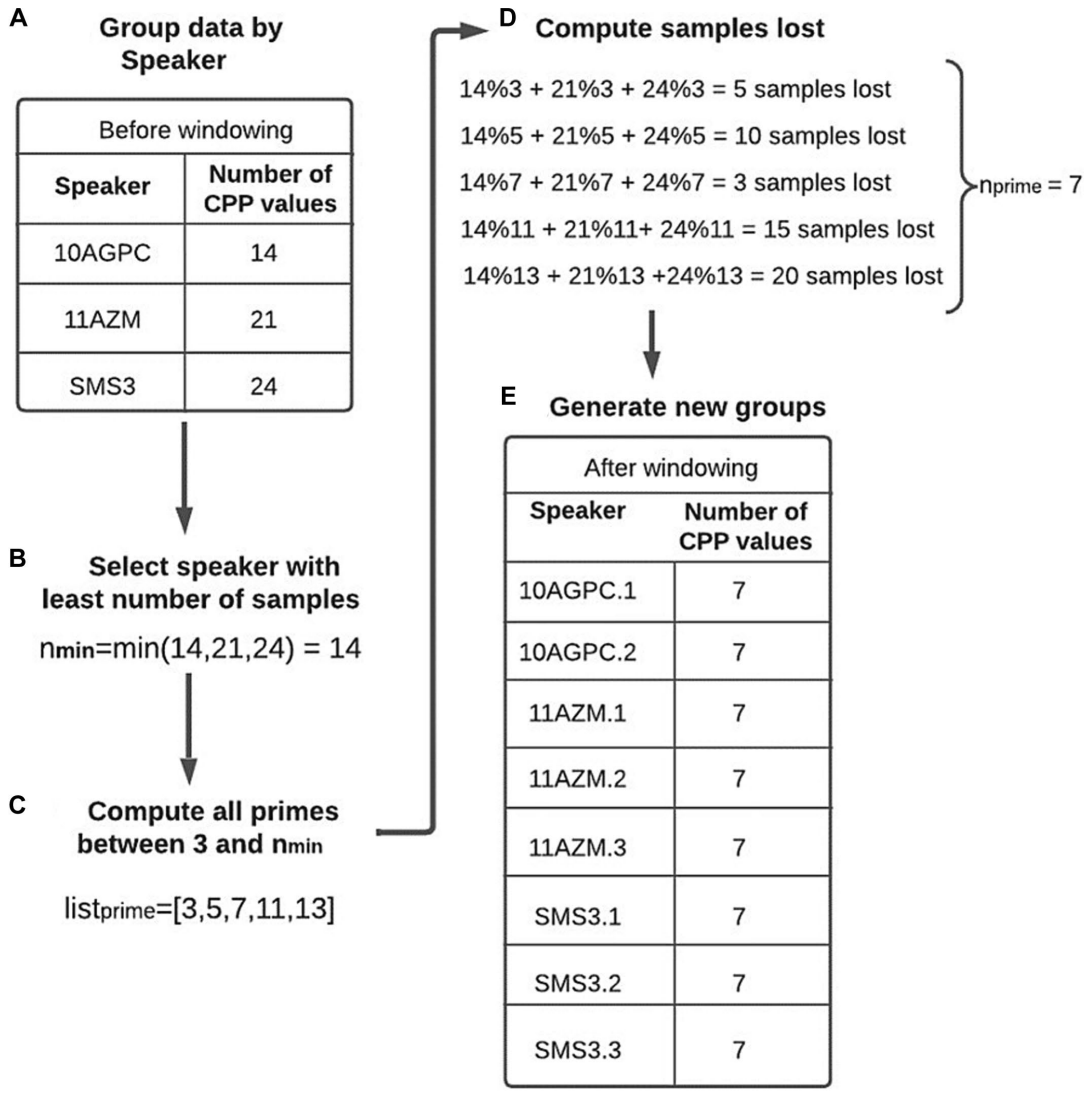
Windowing example. **(A)** All the CPP values stored in the database are grouped for each speaker. In the illustrated example, the first speaker has 14 CPP values, the second has 21 and the third has 24. **(B)** The speaker with the lowest number of samples (14 in this case) is identified ($n_{\text{min}}$). **(C)** All prime numbers between 3 and $n_{\text{min}}$ (14) are stored in a list ($list_{\mathrm{prime}}$). **(D)** For each value stored in $list_{\mathrm{prime}}$, the number of samples that would be lost when dividing the sampling into groups of that size is calculated. This calculation is equivalent to determining the modulus of the group size between that value. Suppose that in the example described above that a value of three is used. Since the first speaker has 14 samples, it is possible to generate four new groups of size three and lose two samples; for the second speaker, no samples would be lost, and for the third speaker, three samples would be lost. Therefore, if a size of three is used to generate the new groups, a total of five samples would be lost. For this reason, an $n_{\mathrm{prime}}$ value is sought that minimizes the number of lost samples. **(E)** The samples grouped by speaker are divided into groups of size $n_{\mathrm{prime}}$. Each subgroup generated from the same individual has a number added to the end of the identifier to distinguish them. In the case of the above example, the number of samples of speaker SMS3 is 24, and $n_{\mathrm{prime}}$ is equal to 7. Therefore, three new groups of size 7 are obtained (SMS3.1, SMS3.2, and SMS3.3), and the remaining three samples are lost.

When working with machine learning models, the data must have adequate structure that guarantees correct training. It should be noted that group the information by speaker does not require that all individuals have the same number of samples (the number of voice recordings). It is also unlikely that the recordings will have the same duration. However, to directly apply one or more of the extracted features, the problem of comparing patterns of different sizes must be solved. Therefore, a proprietary “window” algorithm was developed, and to explain its operation, Figure 3 is used as a reference.

Although there are several subgroups that belong to the same person, they should not be treated independently within the dataset. Consequently, they should be assigned exclusively to either the validation set or the training set, but never simultaneously. Though CPP is not an efficient acoustic measure for speaker identification, compared to others such as Mel Frequency Cepstral Coefficients (MFCC) (Ayvaz et al., 2022), it is preferred to avoid mixing subgroups of the same person in the validation and training sets to pre-vent possible data leakage. Table 1 illustrates the result of the windowing process by means of a dataframe, where each row represents a sample in the dataset. With this process, a usable data structure was achieved to train the different ML models, as detailed in the following section.

**Table 1:** Dataframe generated after windowing when $n_{\mathrm{prime}}$ = 7.

Name	CPP1	CPP2	CPP3	CPP4	CPP5	CPP6	CPP7	Target	Sex	Group
10APG.1	0.0488	0.0502	0.5050	0.0501	0.0494	0.0481	0.0476	N	Female	Older
10APG.2	0.0490	0.0508	0.0467	0.0483	0.0457	0.0458	0.0466	N	Female	Older
...	...	...	...	...	...	...	...	...	...	...
SMS3.3	0.0477	0.0475	0.480	0.0511	0.055	0.058	0.058	SMS	Male	Young

In addition to the problem indicated above, there is a second problem, i.e., the imbalance between the classes to be predicted (246 entries from SMS individuals and 100 entries from normotypic individuals). This fact directly affects the performance of models that tend to overfit. To solve this problem, various solutions have been explored, e.g., assigning a higher weight to the minority class during the training or eliminating majority class samples. Finally, it was decided to use the SMOTE technique (Chawla et al., 2002), an oversampling technique based on the creation of synthetic examples of the minority class. With SMOTE, new samples are introduced along the segments that join the k nearest neighbors of the minority class. The number of k neighbors selected depends on the number of samples generated samples required. As the number of samples increases, the number of neighbors employed decreases. The great advantage of this technique is that it allows the generation of synthetic samples instead of resorting to oversampling, where samples of the minority class are reintroduced into the dataset, which tends to lead to overfitting.

### 2.4 ML techniques

In this work, both supervised and unsupervised methods were considered to compare the different techniques and create combined models. Among unsupervised methods, the Gaussian mixture model (GMM) (Rasmussen, 1999) and K-means clustering (Sinaga and Yang, 2020) were used. In addition, the following supervised methods were used: SVM, random forest (RF), linear discriminant analysis (LDA) and k-nearest neighbors (KNN).

Unsupervised methods were not included in this work as they do not offer results that contribute any new research knowledge. These techniques generated clusters based on the sex and age of the individuals, ignoring the CPP. Therefore, the experiment was repeated after eliminating these two variables. However, the clusters did not provide any new information.

**Figure 4:**
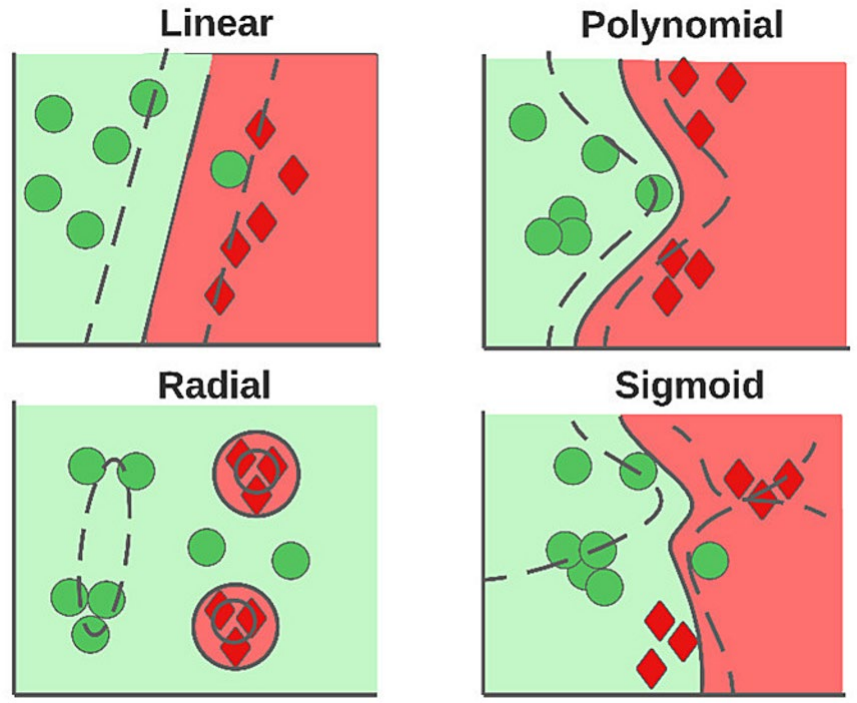
Hyperplanes generated according to the kernel used.

Because supervised techniques are well known, only a brief description of the methods is given. The SVM (Jakkula, 2006) builds hyperplanes that allow an optimal separation of the data, and the power of this method resides in the kernel trick, allowing data transfer to spaces of greater dimensionality in an optimal manner. Depending on the kernel used, the shape of the decision boundary varies; in Figure 4, the influence of the different types of kernels is observed.

The RF (Pachange et al., 2015) is an assembly method, where multiple decision trees are combined to generate predictions. This method is based on building decision trees, where data are divided using the problem variables, applying some criterion that evaluates and maximizes the gain of information. LDA searches for a linear combination of the characteristics that generates the greatest variance between classes and minimizes it within each class (Izenman, 2008). KNN allows for the prediction of a class of data based on its k closest neighbors (Uddin et al., 2022). The way in which the influence of each neighbor is determined in the final prediction can vary according to the technique used. For example, if the weight of each neighbor in the final decision is “uniform,” all neighbors have an equal influence on the vote; on the other hand, if each neighbor is “weighted,” the closest neighbors will have a greater influence on the final decision.

### 2.5 Wilcoxon rank sum test

The Wilcoxon rank-sum test, also called the Mann–Whitney U test, is a powerful tool for comparing two sets of data without relying on specific assumptions about their distribution (unlike some other tests). It works by ranking the observations in each set instead of using their raw values. This makes it especially useful when the data might be skewed or non-normally distributed.

The goal of the Wilcoxon rank-sum test is to assess whether the medians of two populations differ significantly. This is particularly helpful when the precise shape of the data distribution is unknown.

To calculate the test statistic, the formula is shown as follows:


$$U=n_{1}m_{2}+\sum R_{1}-\left(n_{1}\right)\left(n_{2}+1\right)/2$$

Where:

- *U*: The test statistic
- *n₁*: Size of the first sample
- *n₂*: Size of the second sample
- Σ*R₁*: Sum of the ranks in the first sample
- *m₂*: Median of the second sample

## 3 Results

### 3.1 Training and validation

**Figure 5:**
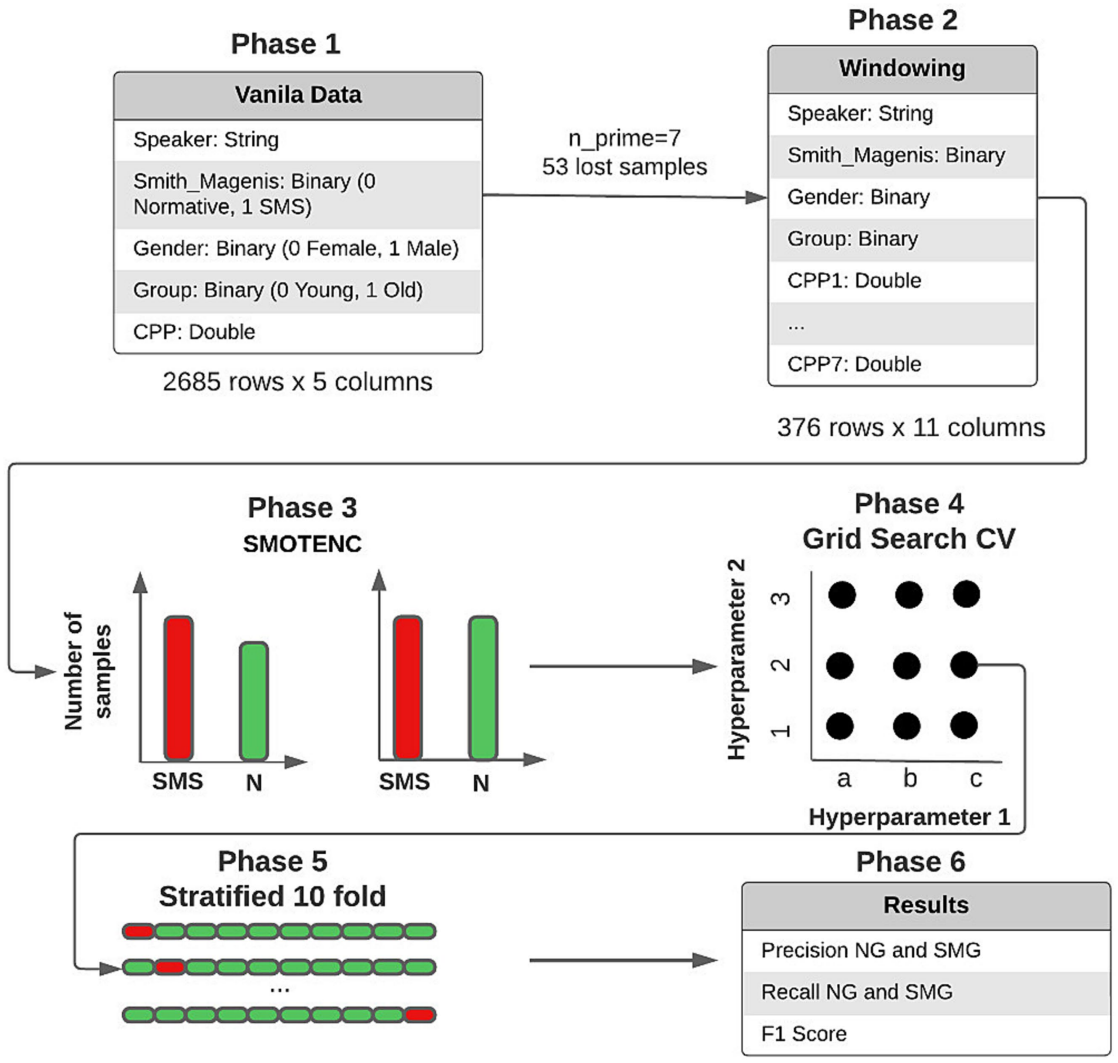
Data preprocessing and obtained results.

The consistency of this study lies in its data, as well as the techniques and methods used. Therefore, it was decided to apply the methodical procedure described in Figure 5 to the data. This procedure is summarized in four fundamental phases: windowing, Leave One Out, SMOTE, ML methods.

1. Windowing: Each sample is composed of seven CPP values, sex, and group. Therefore, $n_{\mathrm{prime}}$ = 7.
2. Leave One Out (LOO): It is used to implement a training and validation model that ensured that different subgroups of the same person do not end up in different datasets. To do this, all subgroups of the same person are extracted to be used as a validation set, while the rest of the samples are used in the training phase. This process is repeated for each of the 24 people in the study.
3. SMOTE: It is used to generate new synthetic samples of the minority class (normotypic). The objective is to avoid creating biased models that tend to over-identify the dominant class (SMS). Although the number of SMS and normative individuals in the training set is always 11 versus 12, depending on which group is used for validation, the number of SMS subgroups (248) is higher than that of normative subgroups (131). It should be noted that this technique is only applied to the training set. The SMOTE technique is not suitable for the validation set. In such a way that the two groups are separated and do not mix and therefore data leakage is avoided.
4. ML methods: Once the training and validation sets are obtained, the different ML models are trained. Previously, exhaustive tests were carried out with different hyperparameters to identify the most effective combinations. It should be noted that, for each validation set, not only one but ten iterations are carried out. An augmented training set is generated in each iteration by using the SMOTE technique. Then, the performance of the used model is evaluated on the validation set. This process is repeated ten times, generating new training sets with SMOTE and training a new model in each iteration. The aim is to obtain a robust and accurate estimate of the model’s performance over iterations. This process consists of a Leave One Out Cross Validation.

To statistically compare the performance of the different models on each individual, the following process will be followed: the 10 values obtained in the LOO for each subject in each method will be recorded. Then, all the results of each method for the same individual will be compared one by one using the Wilcoxon Rank Sum Test (Boslaugh, 2012), in order to obtain the *p*-values of and thus determine the statistical significance of the methods. The results are reflected in Tables 2, 3.

**Table 2:** Summary and comparison of the four ML methods, providing average and pairwise precision rates using the Wilcoxon Rank Sum Test for CE1.

Average accuracy					Comparison Wilcoxon Test (p-value)					
RF	KNN	SVM	LDA	Speaker	RF_vs_SVM	RF_vs_KNN	RF_vs_LDA	SVM_vs_KNN	SVM_vs_LDA	KNN_vs_LDA
59.23%	53.85%	46.15%	61.54%	10AGPC	0.002	0.011	0.149	0.002	0.002	0.002
99.23%	92.31%	100.00%	100.00%	11AAZM	1	0.003	1	0.002	NA	0.002
23.08%	15.38%	0.00%	0.00%	11OADS	0.002	0.005	0.002	0.002	NA	0.002
88.33%	91.67%	75.00%	75.00%	511O	0.002	0.072	0.002	0.002	NA	0.002
16.67%	16.67%	16.67%	16.67%	517A	NA	NA	NA	NA	NA	NA
5.83%	0.00%	0.00%	0.00%	612A	0.011	0.011	0.011	NA	NA	NA
87.14%	71.43%	71.43%	71.43%	618O	0.002	0.002	0.002	NA	NA	NA
49.00%	40.00%	40.00%	40.00%	637A	0.008	0.008	0.008	NA	NA	NA
85.71%	85.71%	85.71%	85.71%	743O	NA	NA	NA	NA	NA	NA
87.06%	94.12%	82.35%	94.12%	819O	0.018	0.012	0.012	0.002	0.002	NA
67.33%	66.67%	46.67%	66.67%	842O	0.002	0.783	0.783	0.002	0.002	NA
95.00%	100.00%	100.00%	100.00%	12109A	0.149	0.149	0.149	NA	NA	NA
99.64%	100.00%	100.00%	100.00%	SMS1	1.000	1.000	1.000	NA	NA	NA
100.00%	100.00%	100.00%	100.00%	SMS2	NA	NA	NA	NA	NA	NA
86.15%	92.31%	92.31%	92.31%	SMS3	0.006	0.006	0.006	NA	NA	NA
100.00%	100.00%	100.00%	100.00%	SMS4	NA	NA	NA	NA	NA	NA
77.50%	87.50%	100.00%	100.00%	SMS5	0.002	0.006	0.002	0.002	NA	0.002
68.46%	76.92%	84.62%	84.62%	SMS6	0.002	0.010	0.002	0.002	NA	0.002
29.23%	46.15%	38.46%	38.46%	SMS7	0.007	0.002	0.007	0.002	NA	0.002
90.61%	87.88%	93.94%	93.94%	SMS8	0.002	0.003	0.002	0.002	NA	0.002
36.15%	15.38%	7.69%	0.00%	SMS9	0.002	0.002	0.002	0.002	0.002	0.002
100.00%	100.00%	100.00%	100.00%	SMS10	NA	NA	NA	NA	NA	NA
62.88%	65.38%	65.38%	61.54%	SMS11	0.026	0.026	0.104	NA	0.002	0.002
91.76%	94.12%	100.00%	100.00%	SMS12	0.002	0.006	0.002	0.002	NA	0.002
71.1%	70.6%	68.6%	70.1%							

**Table 3:** Summary and comparison of the four ML methods, providing average and pairwise precision rates using the Wilcoxon Rank Sum Test for CE2.

Average accuracy					Comparison Wilcoxon test (*p*-value)					
RF	KNN	SVM	LDA	Speaker	RF_vs_SVM	RF_vs_KNN	RF_vs_LDA	SVM_vs_KNN	SVM_vs_LDA	KNN_vs_LDA
72.30%	90.00%	99.23%	89.23%	10AGPC	0.002	0.002	0.002	0.008	0.008	0.679
100.00%	100.00%	100.00%	100.00%	11AAZM	NA	NA	NA	NA	NA	NA
33.80%	33.85%	81.54%	57.69%	11OADS	0.002	1.000	0.002	0.002	0.002	0.002
93.30%	100.00%	100.00%	95.83%	511O	0.006	0.006	0.299	NA	0.037	0.037
21.70%	55.00%	50.00%	35.00%	517A	0.002	0.002	0.015	0.149	0.003	0.002
9.20%	10.00%	8.33%	0.00%	612A	0.414	0.679	0.006	0.186	0.002	0.007
92.90%	100.00%	100.00%	100.00%	618O	0.037	0.037	0.037	NA	NA	NA
56.00%	65.00%	70.00%	88.00%	637A	0.002	0.058	0.002	0.240	0.002	0.002
85.71%	91.43%	100.00%	100.00%	743O	0.002	0.072	0.002	0.020	NA	0.020
84.12%	100.00%	100.00%	100.00%	819O	0.002	0.002	0.002	NA	NA	NA
73.33%	74.00%	98.00%	96.00%	842O	0.002	1.000	0.002	0.002	0.149	0.002
100.00%	100.00%	100.00%	100.00%	12109A	NA	NA	NA	NA	NA	NA
99.64%	98.21%	92.86%	98.93%	SMS1	0.002	0.129	0.424	0.002	0.002	0.484
92.94%	91.18%	94.12%	94.12%	SMS2	0.186	0.322	0.186	0.037	NA	0.037
86.15%	85.38%	76.92%	73.08%	SMS3	0.002	0.408	0.002	0.012	0.037	0.008
100.00%	100.00%	100.00%	100.00%	SMS4	NA	NA	NA	NA	NA	NA
75.00%	70.00%	75.00%	97.50%	SMS5	1.000	0.129	0.002	0.072	0.002	0.002
43.85%	37.69%	27.69%	30.00%	SMS6	0.002	0.098	0.002	0.034	0.149	0.033
14.62%	21.54%	7.69%	23.08%	SMS7	0.048	0.090	0.026	0.002	0.002	0.186
84.24%	77.58%	67.27%	86.67%	SMS8	0.002	0.006	0.229	0.009	0.002	0.002
30.77%	13.08%	0.00%	0.00%	SMS9	0.002	0.002	0.002	0.002	NA	0.002
100.00%	100.00%	100.00%	100.00%	SMS10	NA	NA	NA	NA	NA	NA
56.15%	46.15%	34.62%	44.23%	SMS11	0.002	0.009	0.009	0.002	0.002	0.322
88.53%	88.82%	80.59%	98.24%	SMS12	0.002	1.000	0.002	0.002	0.002	0.002
70.6%	72.9%	73.5%	75.3%							

### 3.2 Results

Two different case studies were established in order to evaluate the behavior and quality of the predictions in the models.

1. The first case study (CE1) applies the windowing process but does not use SMOTE, resulting in an unbalanced training set in favor of the SMS class. Each training/validation sample contains seven CPP values used to predict whether it belongs to the SMS or normative class.
2. The second case study (CE2) involves the data passing through the windowing process and subsequently applying SMOTE to the training set. The data maintains the same structure as in the previous case.

**Figure 6:**
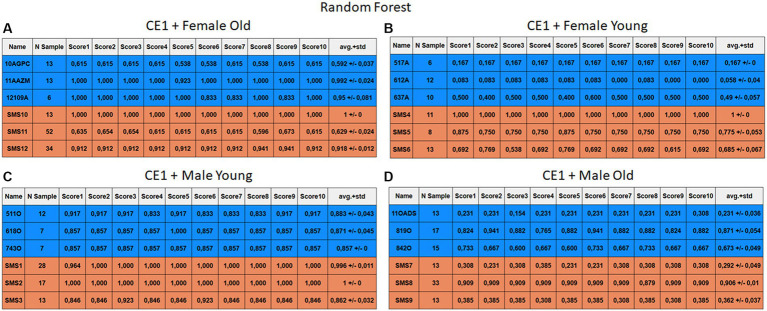
Summary of the results for the CE1, using RF. **(A)** Detailed performance for the old female subgroup. **(B)** Detailed performance for the young female subgroup. **(C)** Detailed performance for the young male subgroup. **(D)** Detailed performance for the old male subgroup.

**Figure 7:**
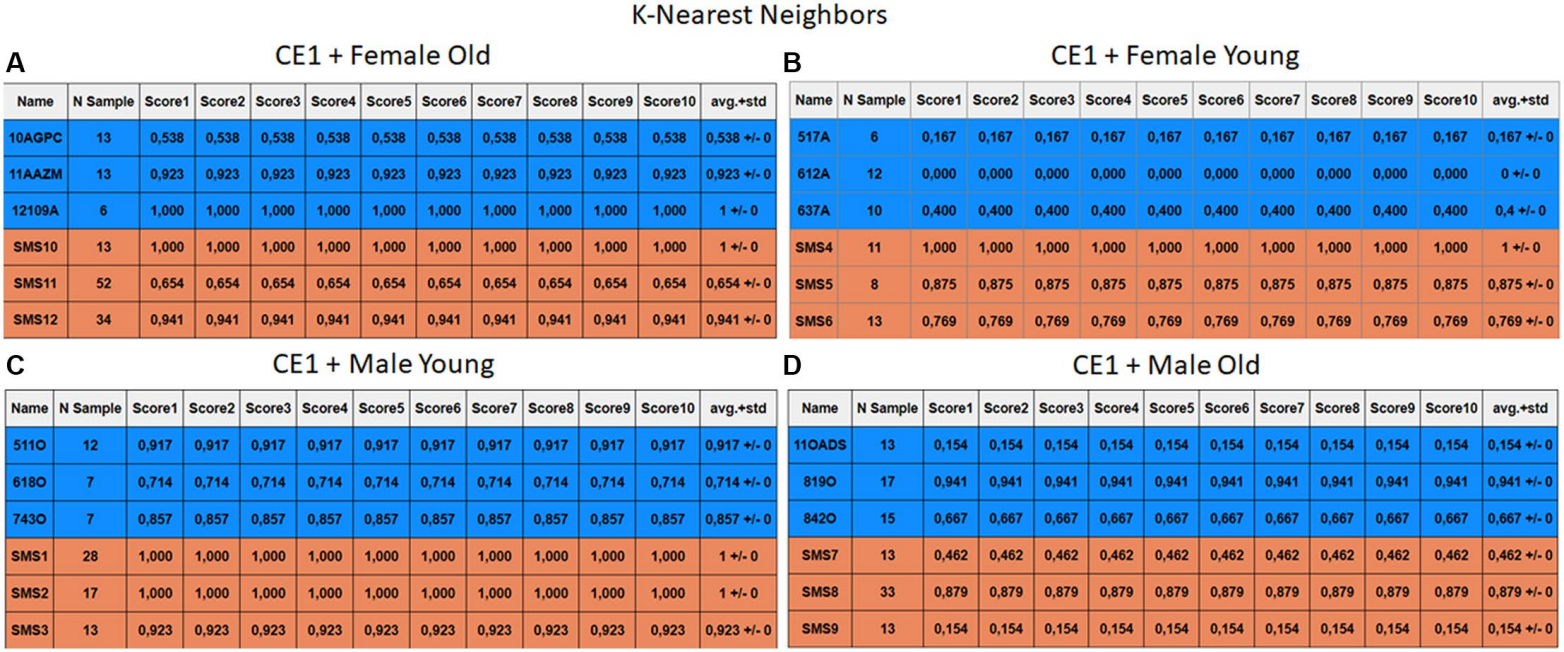
Summary of the results for the CE1, using KNN. **(A)** Detailed performance for the old female subgroup. **(B)** Detailed performance for the young female subgroup. **(C)** Detailed performance for the young male subgroup. **(D)** Detailed performance for the old male subgroup.

**Figure 8:**
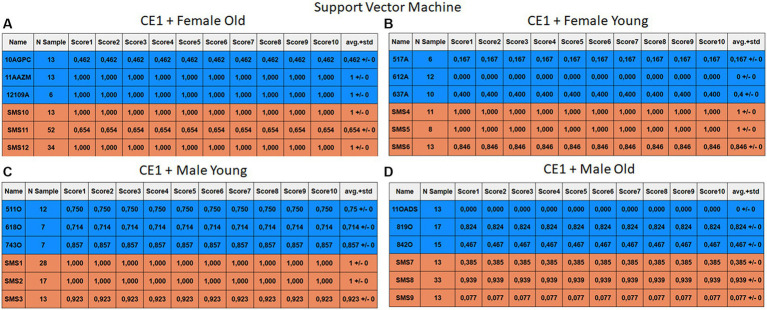
Summary of the results for the CE1, using SVM. **(A)** Detailed performance for the old female subgroup. **(B)** Detailed performance for the young female subgroup. **(C)** Detailed performance for the young male subgroup. **(D)** Detailed performance for the old male subgroup.

**Figure 9:**
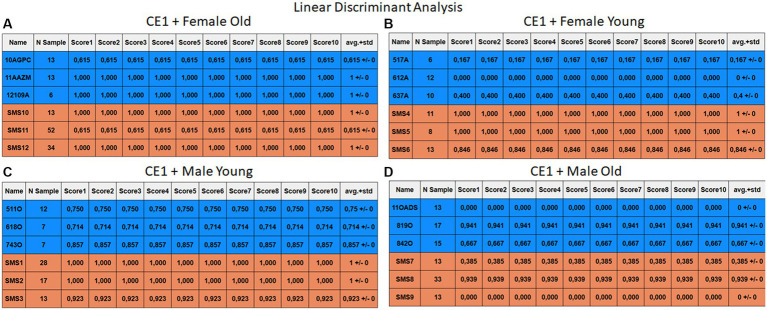
Summary of the results for the CE1, using LDA. **(A)** Detailed performance for the old female subgroup. **(B)** Detailed performance for the young female subgroup. **(C)** Detailed performance for the young male subgroup. **(D)** Detailed performance for the old male subgroup.

**Figure 10:**
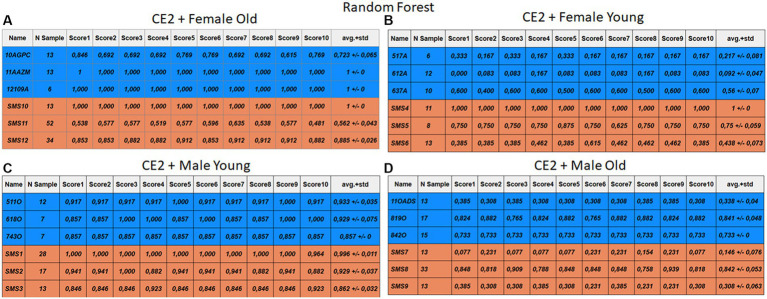
Summary of the results for the CE2, using RF. **(A)** Detailed performance for the old female subgroup. **(B)** Detailed performance for the young female subgroup. **(C)** Detailed performance for the young male subgroup. **(D)** Detailed performance for the old male subgroup.

**Figure 11:**
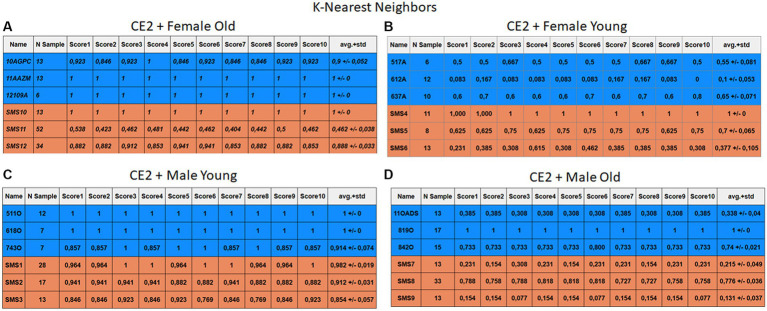
Summary of the results for the CE2, using KNN. **(A)** Detailed performance for the old female subgroup. **(B)** Detailed performance for the young female subgroup. **(C)** Detailed performance for the young male subgroup. **(D)** Detailed performance for the old male subgroup.

**Figure 12:**
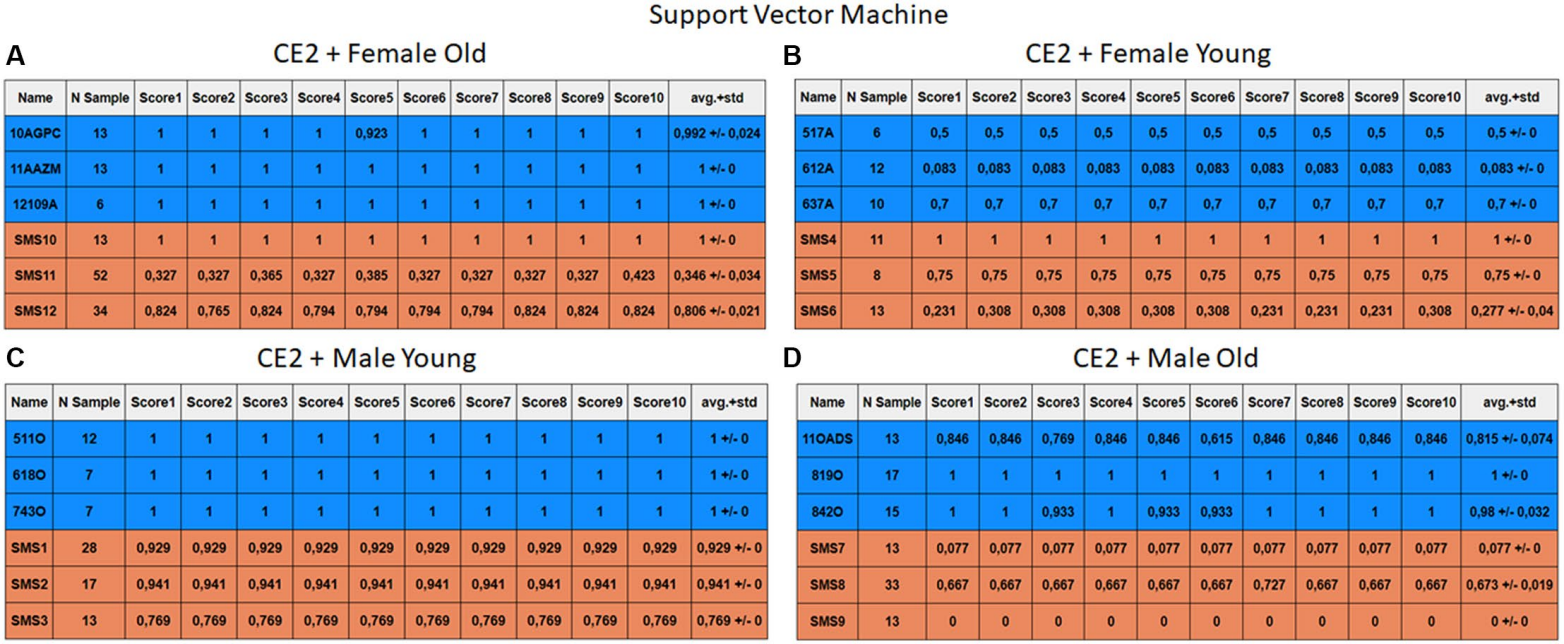
Summary of the results for the CE2, using SVM. **(A)** Detailed performance for the old female subgroup. **(B)** Detailed performance for the young female subgroup. **(C)** Detailed performance for the young male subgroup. **(D)** Detailed performance for the old male subgroup.

**Figure 13:**
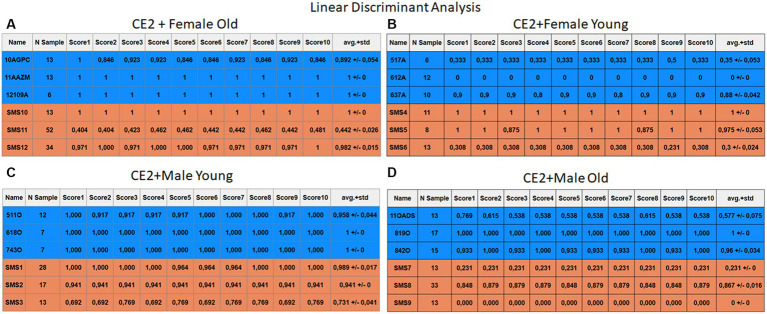
Summary of the results for the CE2, using LDA. **(A)** Detailed performance for the old female subgroup. **(B)** Detailed performance for the young female subgroup. **(C)** Detailed performance for the young male subgroup. **(D)** Detailed performance for the old male subgroup.

Each case relates to the four ML techniques proposed in Section 2.4. Each figure (Figures 6–13) groups individuals by their age, sex, and study case, corresponding to the subgroups identified in Section 2.2. Each figure is divided into tables which share the same column structure: the first identifies the speaker, the second shows the number of samples per person obtained after the windowing process. The next ten columns represent the values obtained using leave-one-out (LOO) cross-validation, with the samples treated as the validation group, these ten values reflect the repetitions of the process. The last column is the average value of the ten iterations plus the standard deviation. Every table displays three normative (blue) and the non-normative (orange) individuals. In each iteration of the Leave-One-Out (LOO) cross-validation, all samples belonging to a single individual are consistently used as the validation set. This means we exclude all samples from a particular subject and test the model on them in each iteration.

Importantly, the tables associated with CE2 (Figures 10–13) exhibit higher standard deviations and different results on the score columns compared to those of CE1 (Figures 6–9). This issue occurs because, in CE2, each iteration augments the training set with SMOTE, generating new synthetic data, making each training set different from the others. Furthermore, significant variation between iterations for the same subject is possible due to the limited size of the individual validation sets (i.e., 15 samples). If the algorithm fails or hits two samples of the available data during a specific iteration, the resulting value for that iteration can fluctuate significantly across different runs.

#### 3.2.1 Case study 1

The results of CE1 are elaborated in Figures 6–9. It is noteworthy that the subgroups of Female Old and Male Young (Figures 6A–9A, 6C–9C) do not exhibit exceptionally low detection rates. However, a stark contrast is observed in the Female Young subgroup (Figures 6B–9B), where the three normative individuals display significantly lower results compared to the SMS group. In the final subgroup, Male Old (Figures 6D–9D), both normative and SMS individuals demonstrate low detection rates.

When individuals are evaluated independently, it is observed that several normative subjects, such as 10AGPC, 11OADS, 517A, 612A, 637A, and 842O, exhibit low precision rates across various methods. Some of these subjects achieve low rates on the order of 0.1%. Within the SMS group, only SMS7 and SMS9 display significantly low detection rates. SMS11 also has a low rate, albeit higher than the previous two speakers. These results align with the tendencies of a biased model, which tends to over-identify the majority groups. In this scenario, the dominant class (SMS) demonstrates better detection than the minority class (normative).

#### 3.2.2 Case study 2

Figures 10–13 depict the outputs of CE2. In Figures 10A–13A, there is a noticeable enhancement in the detection of 10AGPC compared to the previous case, notwithstanding with a minor decline for SMS11. In the Female Young subgroup (Figures 10B–13B), detection rates for subjects 517A and 637A have increased, but performance for patient SMS06 has decreased. In the Male Old subgroup (Figures 10D–13D), all normative subjects exhibit improvements in their detection rates, despite a minor decrease for subjects SMS07 and SMS08. Lastly, the Male Young subgroup (Figures 10C–13C) mirrors the Male Old, with improved detection for all normative individuals and a slight decrease for SMS.

Highlighting some individual cases, it is significant to note that subjects 10AGPC and 842O from the normative set have seen substantial improvements in their detection compared to the previous case. The individual 11OADS depicts a considerable increase in SVM detection from 0 to 0.815 (Figures 8D vs. 12D) and an increase from 0 to 0.577 in LDA (Figures 9D vs. 13D). For 637A (Female Young), there is a global enhancement in detection across methods, with both SVM and LDA (Figures 12B, 13B) yielding favorable results. However, no significant improvement is observed for subjects 517A and 612A (Female Young). Conversely, the SMS group results indicate a marked decrease in performance, especially for individuals SMS6 (Female Young) and SMS11 (Female Old), which achieved identification rates below 0.5. SMS7 (Male Old) and SMS9 (Male Old) present identification rates comparable to the previous case. Lastly, the SMOTE technique boosts the precision rates of the minority class, albeit at a slight detriment to the majority class.

## 4 Discussion

In this work, we propose the development of ML models that allow for the identification of SMS versus normotypic individuals. One clinical feature of the SMS pathology is voice hoarseness (Elsea and Girirajan, 2008), as described in previous studies (Hidalgo-De la Guía et al., 2021b), it has been demonstrated that by utilizing the CPP values of SMS and normotypic individuals, it is possible to create divisions into highly differentiated subgroups. This differentiation is primarily due to the hoarseness present in individuals with this genetic pathology. These types of studies are necessary to improve early disease detection. Currently, the average SMS diagnosis age is approximately seven years (Hidalgo-De la Guía et al., 2021b), leading to problems for these patients. Problem arises because SMS requires specific therapies that, when implemented late, cause different kinds of delays. As presented in this research work, the voice is a versatile, inexpensive, and minimally invasive medium that helps to discriminate possible pathologies (Jeffery et al., 2018; Lee, 2021; Calà et al., 2023).

The initial data were not suitable for ML model training. The main problem was sample imbalance between groups. Two techniques were proposed to solve this problem. The first technique is CPP sample “windowing,” a novel approach. In Section 2.3, it was explained that “windowing” consists of grouping the samples by speaker and making new subgroups of the same size to solve the sample imbalance problem. The second technique is the application of SMOTE, with which new synthetic samples of the minority class are generated until a balance between the two classes is achieved. The authors maintain that, with the combination of the “windowing” and SMOTE methods, the dataset is improved. To demonstrate how the yields of the models vary according to the applied techniques, two different case studies were proposed.

The LOO technique was implemented to prevent the inclusion of subgroups of the same person in the validation and training sets, avoiding the risk of data leakage. This technique is especially beneficial in small datasets because it allows the use of all n-1 available data for training. It should be noted that training involves the 23 individuals present in the dataset, while the remaining person is reserved for validation. This validation and training process is iterated ten times for each speaker. This iterative approach contributes to obtaining robust results, reducing the possibility of achieving biased or circumstance-influenced performances. The different models tend to over-identify the dominant group (SMS) in CE1. In contrast, in CE2, the SMOTE technique was implemented in the training dataset to address the class imbalance. It should be highlighted that the application of SMOTE was limited to the training set to prevent possible data leakage.

This approach increased the identification of the normative group and led to an overall improved performance but reduced slightly the identification of the SMS speakers. To evaluate the ML techniques against each other, it has been decided to give the arithmetic median obtained in the SMS and normative classes, as it is not affected by outsider high or low performances in certain individuals. Firstly, SVM offered the worst results, especially in CE1, since it was necessary to use models with a hyperparameter configuration that tends to overfit the model due to its inability to detect the normative class. This led to labeling all results as SMS, obtaining an average of 0.59 and 0.97 for the normative and SMS classes. However, in CE2, a model that does not depend on hyperparameters is obtained, with a median of 0.99 for normative and 0.75 for SMS. In this second case study, its high detection rate in the normative group stands out. Individual 11OADS is far superior to the rest of the methods. Nonetheless, it is not able to achieve such good generalization in the SMS group.

The second model discussed in this study is RF. Acceptable performance is achieved with medians of 0.765 for normative and 0.884 in SMS at CE1. However, practically identical performance is observed to the previous case in CE2. Medians are between 0.787 and 0.852 for normative and SMS. It is crucial to say that the use of SMOTE does not always guarantee an improvement in model performance. In fact, it can become a problem by generating noise in situations of high dimensionality. Nevertheless, it does not rule out the possibility that the combination of the SMOTE technique with RF can improve results with other datasets. For example, in Abdar et al. (2019) four variants of DTs are proposed to predict coronary artery disease. The article proposes a multi-filtering approach based on supervised and unsupervised methods to modify the weights of the attributes, leading to a 20–30% improvement in the methods.

The two final models analyzed in this study exhibit relevant high performances. Firstly, the KNN’s performance experiences a significant improvement: from medians of 0.69 and 0.9 in CE1 to 0.90 and 0.81 for normative and SMS in CE2. This improvement can be attributed to the data arrangement, as shown in Figure 2, where three out of four clusters present adequate separation. Consequently, this technique is better than the others because if the closest samples are selected then higher recognition rate are obtained. Finally, the model that yields the best results is LDA, with medians of 0.690 and 0.970 for CE1 in normative and SMS, respectively. It is accomplished medians between 0.95 and 0.90 in CE2, making it the model with the most outstanding results throughout the research work.

Tables 2, 3 present a statistical comparison using the Wilcoxon Rank Sum test to evaluate the performance of the four employed ML methods which present the following structure. Each table is divided into three concepts. On the left side, the authors detail the accuracy rates for every ML method (RF, KNN, SVM and LDA) for each subject. The next column provides the speaker identifier. Finally, on the right-hand side, the authors detail the comparisons, contrasting the results obtained in the ten iterations (e.g., RF_score1_ … RF_Score10_) of each method against the ten iterations (e.g., LDA_score1_ … LDA_Score10_) of another method for the same subject. The last six columns display p-values from the Wilcoxon test. A *p*-value less than or equal to 0.05 indicates statistically significant differences in accuracy rates between methods, leading to rejection of the null hypothesis that they are equal. The table occasionally shows “Not Applicable (NA)” values. This occurs when the Wilcoxon test cannot calculate a p-value because the distance between all elements of the two input methods is zero. Such scenarios mostly arise when both methods achieve 100% or 0% accuracy (particularly in Table 3) but can also occur with other values. It is likely due to the relatively small dataset size (6–13 samples per subject), which increases the chance of different models achieving identical performance.

Upon comparing the two Tables 2, 3, a disparity is observed in the number of NA values. Table 2 records 59 NA values (29 in normotypic group and 30 in non-normotypic group). Indeed, the Table 3 shows 34 NA values (20 in normotypic group and 14 in non-normotypic group). This difference can be attributed to the limitation of the training dataset in CE1 (without SMOTE), which leads to the models generating identical results due to data bias. However, when SMOTE is applied, the different models can produce diverse results due to data augmentation process and the correction of bias during training. Analyzing the results reveals that some speakers, like 11AAZM and SMS04, are highly identifiable across all methods, achieving 100% accuracy and received “Not Applicable” (NA) values in all one-to-one Wilcoxon comparisons. Likewise, while most comparisons yield p-valuess below 0.05, indicating statistically significant differences, the RF vs. KNN comparison shows 12 non-significant results. This suggests similar performance for these methods, potentially making them less effective than the others. Conversely, SVM and LDA generally exhibit more statistically significant values, implying stronger distinctions in their performance compared to the other ML methods.

Another point of debate is whether the SMOTE technique can affect the performance of the different models. In Blagus and Lusa (2013), the authors applied this technique to high-dimensionality cases. However, here, it is addressed a single dimension (the CPP). The obtained results agree with those of the previously referenced work. First, the authors noted that for low-dimensionality cases, SMOTE usually represents an improvement (e.g., the RF, SVM and KNN cases) or equates the results to those of other undersampling techniques (e.g., the LDA case). These results agree with those achieved in the current study, i.e., for the four ML techniques used, the results were improved with the application of the SMOTE technique. There are techniques that can be regarded as more beneficial than others while others may be less beneficial (e.g., high-dimensionality cases). For example, a secondary effect of SMOTE is that the new samples from the minority class exhibit variances one-third smaller than those of the original distribution. This result implies that this technique is not as effective in methods that use variance as an indicator, such as the LDA. RF, SVM, and KNN are the methods that offer better results in cases of low dimensionality. In the case of SVM, it has meant an improvement, but it has not quite reached the expected performance. The reason for this behavior may be due to the combination of the increase in the dimensionality of the SVM itself along with the use of SMOTE. Likewise, the interaction between LDA and KNN methods with SMOTE is negligible, since the Euclidean distance between the classes is the same, before and after the use of SMOTE with low dimensionality, as demonstrated by Blagus and Lusa (2013).

Interestingly, in this research work, the average accuracy across ML methods is similar for every single method. In CE1 (without applied SMOTE technique – see Table 2), all methods achieved values: RF (71.1%), KNN (70.6%), SVM (68.6%), and LDA (70.1%). Notably, RF performed best with 71.1% accuracy.

For CE2 (with SMOTE technique – see Table 3), average accuracy increased across all methods compared to CE1, reaching 70.6% for RF, 72.9% for KNN, 73.5% for SVM, and 75.3% for LDA. Notably, LDA emerged as the best performer in CE2 with an average accuracy of 75.3%. This finding suggests that the data augmentation techniques used in CE2 led to overall improved performance.

## 5 Conclusion

**Figure 14:**
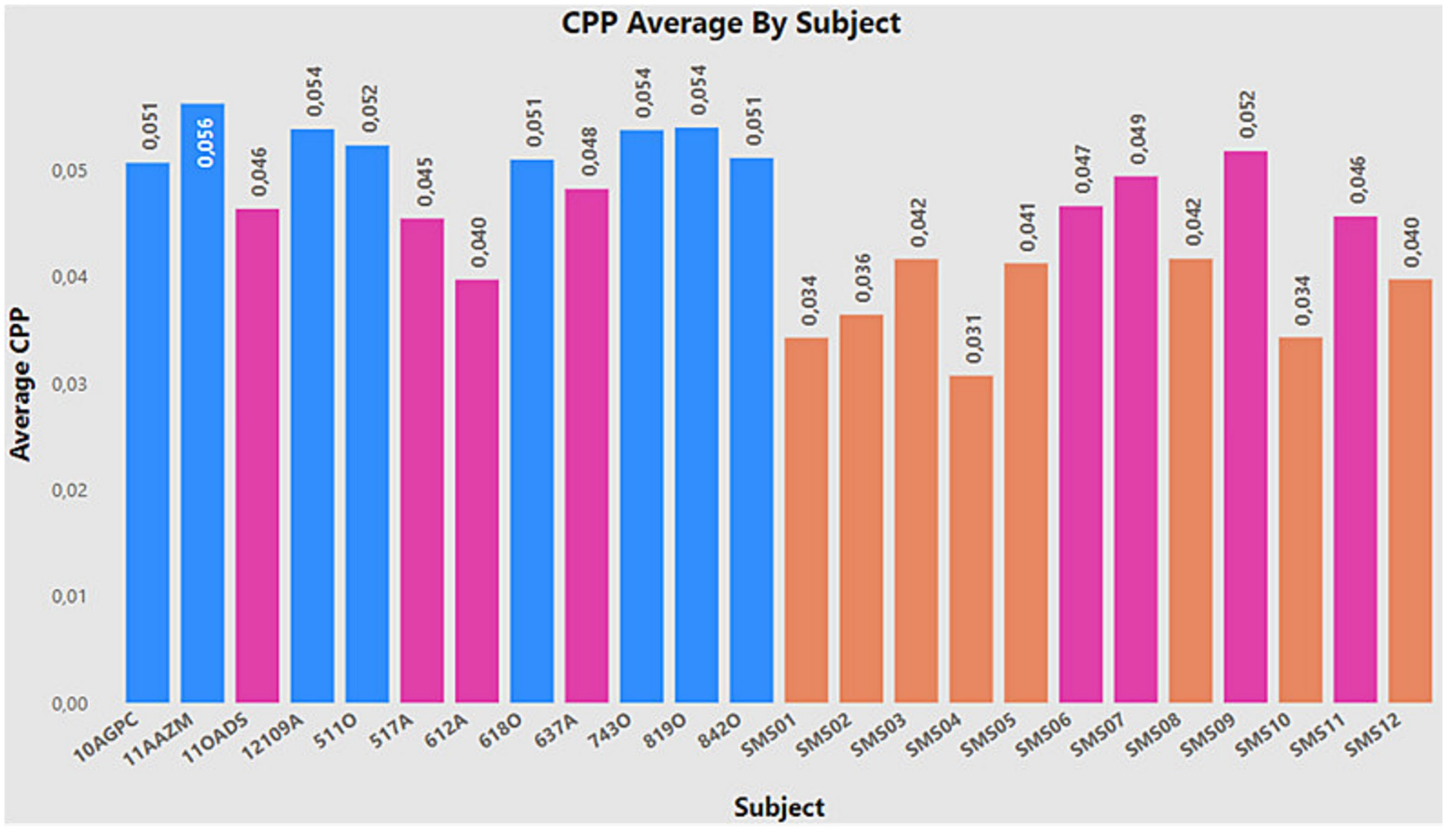
CPP average by subject.

Two objectives have been achieved in the work. The first objective showed that, due to the application of correct data preprocessing, the performance of the models can be improved, as demonstrated through different case studies. Furthermore, the outcomes of CE2 are more reliable and robust compared to the results of CE1, owing to the application of data augmentation techniques. While it may appear that CE1 has a superior classification rate, this is primarily due to the class imbalance, with a greater number of SMS samples compared to normotypical ones. The second goal of the work was to study whether the CPP is a suitable metric for the identification of SMS vs. normotypic individuals, and, according to the results obtained in the last case study, it can be confirmed that this metric fulfils this function. The main limitation of the study is the number of individuals with SMS currently available. However, this situation opens the opportunity to explore different data augmentation methods and compare their performance to find the most suitable one for the study context. A similar process will be carried out with the machine learning algorithms, using different variants of them. Another interesting approach would be the inclusion of cost-sensitive algorithms. As explained in Figure 14, individuals with outlier values have been identified compared to their respective groups. Therefore, it may be beneficial to implement counterfactual methods to decrease the biased caused by those outliers.

Regarding the supervised learning models used, no attempts were made to identify the ideal iteration that would yield a very high result. This is because when such a model is applied in a real-world context, it tends to underperform due to its adaptation to a specific data combination for achieving the results. As a result, the initial case study reveals models that are biased toward the target class (SMS), while the final case study presents models with less bias and a high precision rate. The results also indicate that performance improves following a series of transformations on complex initial data. However, to enhance and solidify these results, it is essential to obtain samples from new subjects.

Furthermore, it is important to highlight the presence of certain individuals who show significantly low detection rates in most models, considering CE2 as a reference. These individuals include 11OADS, 517A, 612A, 637A (the latter shows good performance in LDA and SVM, but not in the rest), as well as SMS6, SMS7, SMS9, and SMS11. Figure 14 presents the average CPP value for everyone stored in the database, remembering that the normative group should exhibit higher CPP values, while the non-normative group should show lower values. The bars marked in pink correspond to the individuals mentioned above, showing how they present higher or lower values than their respective groups. In other words, these individuals constitute the decision boundary of the problem. This finding raises possible future approaches, such as the application of synthetic data augmentation methods on the decision boundary, assigning weights to the problem samples, opening new possibilities to improve model performance.

Finally, two potential avenues of research are proposed. The first involves replicating the same machine learning procedures with other rare diseases, such as WS. The goal would be to compare performance and potentially conduct a case study where different models are trained to distinguish between SW and SMS individuals, thereby extracting the similarities and differences between both pathologies. The second avenue of research would focus on the application of deep learning techniques. However, to develop more robust models, it would first be necessary to increase the number of SMS samples. It should be noted that authors explore several new methods based on SMOTE techniques and data augmentation methods in future research works.

## Data availability statement

The datasets presented in this article are not readily available because the data collected in this research are subject to data protection law due to their biometric and sensitive nature. Furthermore, the study population is minors. Requests to access the datasets should be directed to DP-A, daniel.palacios@urjc.es.

## Ethics statement

The studies involving humans were approved by Universidad Politécnica de Madrid. The studies were conducted in accordance with the local legislation and institutional requirements. Written informed consent for participation in this study was provided by the participants’ legal guardians/next of kin.

## Author contributions

RF-R: Conceptualization, Formal analysis, Software, Writing – original draft, Writing – review & editing. EN-V: Investigation, Supervision, Writing – original draft, Writing – review & editing. IH-d: Data curation, Validation, Writing – review & editing. EG-H: Data curation, Validation, Writing – review & editing. AÁ-M: Supervision, Validation, Writing – review & editing. RM-O: Formal analysis, Software, Writing – review & editing. DP-A: Conceptualization, Funding acquisition, Project administration, Writing – original draft, Writing – review & editing.
